# Supplementary material for: Transport of biodeposits and benthic footprint around an oyster farm, Damariscotta Estuary, Maine
Source: PeerJ. 2021 Aug 11;9:e11862. doi: 10.7717/peerj.11862 (PMC8364327; doi:10.7717/peerj.11862)
Supplement: Supplemental Information 2 — Blank cells represent no data. *very close to zero. [file peerj-09-11862-s002.docx]

| **Site** | **Depth**  **(cm)** | **Water Content** | **Organic matter content and quality** | | | | **Grain size classes** | | | | | **Folk and Ward 1957 description** |
| --- | --- | --- | --- | --- | --- | --- | --- | --- | --- | --- | --- | --- |
|  |  |  | **%OC** | **C:N** | **N**  **(mmol g^-1^)** | **C**  **(mmol g^-1^)** | **% gravel** | **%sand** | **%mud** | **%clay** | **%silt** |  |
|  | 1 | 0.5 | 3.09 |  |  |  | 0.01 | 0.65 | 0.34 | 0 | 0.34 | very fine sand |
|  | 2 | 0.5 | 2.12 | 11.21 | 0.11 | 1.25 | 0 | 0.56 | 0.44 | 0.01 | 0.43 | very coarse silt |
| Away | 3 | 0.55 | 3.6 |  |  |  | 0 | 0.58 | 0.42 | 0.01 | 0.42 | very fine sand |
|  | 4 | 0.6 | 5.53 |  |  |  | 0 | 0.41 | 0.59 | 0.01 | 0.58 | very coarse silt |
|  | 5 | 0.61 | 5.47 |  |  |  | 0 | 0.45 | 0.55 | 0.01 | 0.54 | very coarse silt |
|  | 7 | 0.61 |  |  |  |  | 0 | 0.45 | 0.55 | 0.01 | 0.54 | very coarse silt |
|  | 1 | 0.43 | 1.36 |  |  |  | 0.01 | 0.46 | 0.52 | 0.01 | 0.51 | very coarse silt |
|  | 2 | 0.44 | 1.84 | 11.49 | 0.09 | 1.05 | 0* | 0.37 | 0.63 | 0.01 | 0.63 | very coarse silt |
|  | 3 | 0.46 | 2.19 |  |  |  | 0* | 0.52 | 0.48 | 0.01 | 0.47 | very coarse silt |
| Farm | 4 | 0.52 | 3.05 |  |  |  | 0* | 0.66 | 0.33 | 0 | 0.32 | very fine sand |
|  | 5 | 0.61 | 5.53 |  |  |  | 0* | 0.37 | 0.63 | 0.01 | 0.62 | very coarse silt |
|  | 7 | 0.59 |  |  |  |  | 0* | 0.48 | 0.52 | 0.01 | 0.51 | very coarse silt |
|  | 9 | 0.64 |  |  |  |  | 0* | 0.48 | 0.52 | 0.01 | 0.51 | very coarse silt |
