# Supplementary material for: Transport of biodeposits and benthic footprint around an oyster farm, Damariscotta Estuary, Maine
Source: PeerJ. 2021 Aug 11;9:e11862. doi: 10.7717/peerj.11862 (PMC8364327; doi:10.7717/peerj.11862)
Supplement: Supplemental Information 3 — Due to equipment malfunction during the second incubation (2a), it was repeated (2b) to gather oxygen flux data. Positive values indicate flux out of the sediment and all units are in mmol m−2 d−1. *no data. [file peerj-09-11862-s003.docx]

| **Incubation** | **Site** | **Replicate** | **DIC Flux** | **NH_4_^+^ Flux** | **PO_4_^-3^ Flux** | **Oxygen Flux** |
| --- | --- | --- | --- | --- | --- | --- |
| 1 | Away | 1 | 37.23 | 1.66 | 0.13 | -53.71 |
|  |  | 2 | 72.93 | 2.42 | 0.40 | -34.48 |
|  | Farm | 1 | 86.58 | 0.87 | 0.30 | -58.98 |
|  |  | 2 | 127.57 | 4.20 | 0.56 | -72.53 |
|  | Amended | 1 | 63.12 | 2.76 | 0.54 | No O2 trend |
|  |  | 2 | 54.22 | 2.11 | 0.60 | No O2 trend |
| 2a | Away | 1 | 253.89 | 4.91 | * | * |
|  |  | 2 | 86.27 | 3.05 | 0.49 | * |
|  | Farm | 1 | 98.12 | * | 0.11 | * |
|  |  | 2 | 132.36 | 4.40 | * | * |
|  | Amended | 1 | 132.04 | 4.91 | 0.33 | * |
|  |  | 2 | 149.08 | 3.84 | 0.54 | * |
| 2b | Away | 1 | * | 1.80 | 1.77 | -60.60 |
|  |  | 2 | * | 3.50 | 0.66 | -40.90 |
|  | Farm | 1 | * | 2.59 | 0.24 | -26.50 |
|  |  | 2 | * | 3.38 | 0.01 | -53.84 |
|  | Amended | 1 | * | 2.50 | 0.83 | No O2 trend |
|  |  | 2 | * | 4.75 | 0.21 | No O2 trend |
| **Averaged**  **Fluxes** | Away  Farm  Amended | | 113 ± 96  111 ± 22  100 ± 48 | 2.9 ± 1.2  3.1 ± 1.4  3.5 ± 1.2 | 0.7±0.6  0.2 ± 0.2  0.5±0.2 | -47 ± 11  -53 ± 19  * |
