# Supplementary material for: Transport of biodeposits and benthic footprint around an oyster farm, Damariscotta Estuary, Maine
Source: PeerJ. 2021 Aug 11;9:e11862. doi: 10.7717/peerj.11862 (PMC8364327; doi:10.7717/peerj.11862)
Supplement: Supplemental Information 4 — Numbers in parentheses indicate standard deviation. Letters beside family names indicate phylum/subphylum (M = Mollusca, A = Annelida, C = Crustacea). Numbers are rounded to nearest whole number. [file peerj-09-11862-s004.docx]

| **Family** | **Individuals m^-2^** | |
| --- | --- | --- |
|  | **Farm (*n*=4)** | **Away (*n*=8)** |
| Mactridae (M) | 42 (46) | 28 (39) |
| Myidae (M) | 111 (136) | 56 (62) |
| Mytilidae (M) | 14 (24) | 0 |
| Pharidae (M) | 14 (24) | 35 (39) |
| Yoldiidae (M) | 28 (48) | 14 (24) |
| Capitellidae (A) | 0 | 14 (37) |
| Flabelligeridae (A) | 0 | 125 (198) |
| Nephtyidae (A) | 28 (48) | 3000 (3559) |
| Nereididae (A) | 14 (24) | 14 (24) |
| Orbiniidae (A) | 28 (28) | 0 |
| Spionidae (A) | 167 (289) | 97 (218) |
| Corophiidae (C) | 14 (24) | 28 (39) |
| **Total** | **459 (397)** | **3411 (3902)** |
