# Supplementary material for: Transport of biodeposits and benthic footprint around an oyster farm, Damariscotta Estuary, Maine
Source: PeerJ. 2021 Aug 11;9:e11862. doi: 10.7717/peerj.11862 (PMC8364327; doi:10.7717/peerj.11862)
Supplement: Supplemental Information 15 — Contains files with velocity data from the ADV, salinity and oxygen data from a the onset hobo DO loggers, and data from the YSI instruments. A metadata file is included. [file peerj-09-11862-s015.zip › HydrographicData/HydrographicMetadata.rtf]

MetadataData can be found at: https://drive.google.com/drive/folders/1sSeqRgK_ERrjtymCbcXqJEmpxTRQdmbkDUE: Damarischotta Upper Estuary LOBO surface buoy.  All measurements are from the surface and were taken approximately 1-1.5m below the surface in 14-24ft of water (mean ~5.8m)for details see: http://maine.loboviz.com/loboviz/contact person in Damian’s Lab: Cheyenne, cheyenne.adams@maine.eduA bottom platform was deployed near the center of the Mook oyster farm.Location: 44 deg 00.016 N, 69 deg 32.650 WSensors (on platform unless stated):- DADV: ADV deployed at 37cm above the surface, the body was pointing 228 deg, and the sensor was 138, dominate flow direction was 226- HDO: HOBO DO sensor- Y1: YSI 6600- Y2: YSI 6600- HSF: HOBO conductivity sensor deployed at the surface above the platform ~.5m below surfaceSensor deployed ~90m south of the farm for a control:YC: YSI 6600HSC: HOBO conductivity ~.5m below surfaceTerms:t: time (number, UTC)time: time (datnum, UTC)tem: temperaturesal: salinityden: densityOmg: DO (mg/l)Op: DO (%)Oml: DO (ml/l)n: velocity northe: velocity eastz: velocity upu: flow along major axis (+ flood, - ebb)v: flow along minor axis (perpendicular to u, + rightward when entering estuary from sea)H: depthwl: water level (normalized from H)E: Turbulent Kinetic Energy
